# Supplementary material for: Serotonin neuromodulation directs optic nerve regeneration
Source: Development. 2025 Jul 7;152(13):dev204334. doi: 10.1242/dev.204334 (PMC12276806; doi:10.1242/dev.204334)
Supplement: Supplementary information [file develop-152-204334-s1.pdf]

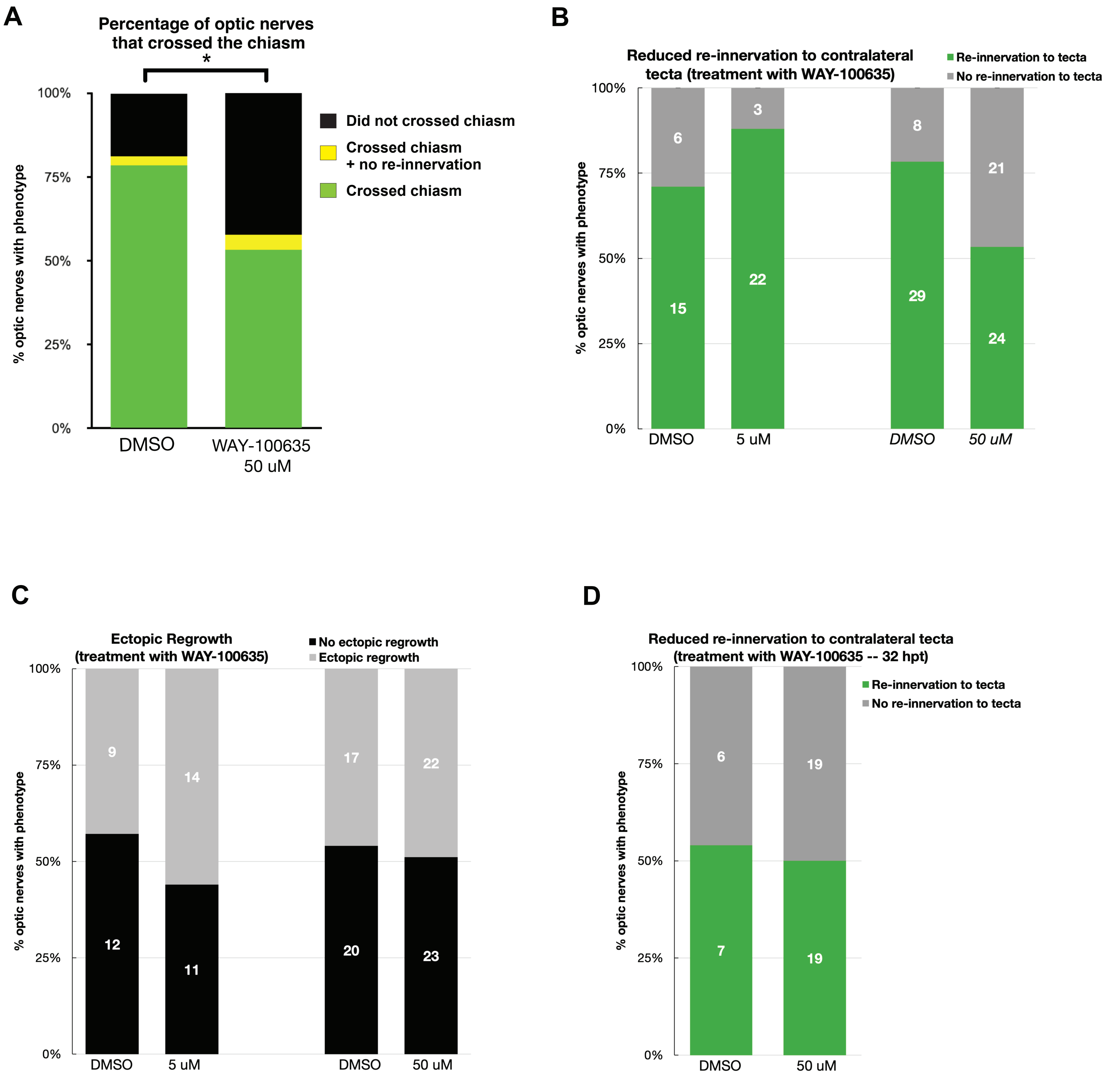

Development • Supplementary information

Fig. S1. Blocking 5-HT1 receptors significantly inhibited the regrowth of optic nerves to the optic chiasm.

(A) Stacked bar graphs showing the percentage of optic nerves that crossed the midline by 48 hpt in Tg(Isl2b:GFP) larvae treated at 24 hpt with DMSO 0.3% or 50 uM of the antagonist WAY-100635. Green bars show the percentage of optic nerves that crossed the chiasm and re-innervated the contralateral tectum. Black bars show the percentage of optic nerves that did not cross the chiasm and did not re-innervated the contralateral tectum. Yellow bars show the percentage of optic nerves that crossed the chiasm but did not re-innervated the contralateral tectum. Data displayed was obtained from the same experimental groups analyzed in Figure 2D. n=37 and n=45 for nerves treated with DMSO and 50 uM WAY-100635, respectively. Statistics determined using the two-tailed Fisher's exact test. Asterisk in figures represent statistical significance: * $P < 0.05$; ns, not significant.

(B) Stacked bar graphs showing the percentage of optic nerves that re-innervated or did not re-innervated the contralateral tecta by 48 hpt in (TgIsl2b:GFP) larvae treated at 24 hpt with DMSO 0.3%, 5 uM of the antagonist WAY-100635 and 50 uM of WAY-100635. Green bars show the percentage of optic nerves that re-innervated the contralateral tectum. Black bars show the percentage of optic nerves that did not re-innervated the contralateral tectum. Numbers inside the bars correspond to the number of optic nerves with a given phenotype. Data displayed was obtained from the same experimental groups analyzed in Figure 2D. n=21 and n=25 for nerves treated with DMSO and 5 uM WAY-100635, respectively; n=37 and n=45 for nerves treated with DMSO and 50 uM WAY-100635, respectively. Please refer to Figure 2D for details on statistical significance. Italicized text highlights groups showing statistical significance.

(C) Stacked bar graphs showing the percentage of optic nerves with ectopic regrowth by 48 hpt in (TgIsl2b:GFP) larvae treated at 24 hpt with DMSO 0.3%, 5 uM of the antagonist WAY-100635 and 50 uM of WAY-100635. Black bars show the percentage of optic nerves with no ectopic regrowth. Gray bars show the percentage of optic nerves with ectopic regrowth. Numbers inside the bars correspond to the number of optic nerves with a given phenotype. Data displayed was obtained from the same experimental groups analyzed in Figure 2E. n=21 and n=25 for nerves treated with DMSO and 5 uM WAY-100635, respectively; n=37 and n=45 for nerves treated with DMSO and 50 uM WAY-100635, respectively. Please refer to Figure 2E for details on statistical significance. Italicized text highlights groups showing statistical significance.

Development • Supplementary information

(D) Stacked bar graphs showing the percentage of optic nerves that re-innervated or did not re-innervated the contralateral tecta by 48 hpt in (TgIsl2b:GFP) larvae treated at 32 hpt with DMSO 0.3% and 50 uM of the antagonist WAY-100635. Green bars show the percentage of optic nerves that re-innervated the contralateral tectum. Black bars show the percentage of optic nerves that did not re-innervated the contralateral tectum. Numbers inside the bars correspond to the number of optic nerves with a given phenotype. Data displayed was obtained from the same experimental groups analyzed in Figure 2F. n=21 and n=25 for nerves treated with DMSO and 5 uM WAY- 100635, respectively; n=13 and n=38 for nerves treated with DMSO and 50 uM WAY-100635, respectively. Please refer to Figure 2F for details on statistical significance. Italicized text highlights groups showing statistical significance.

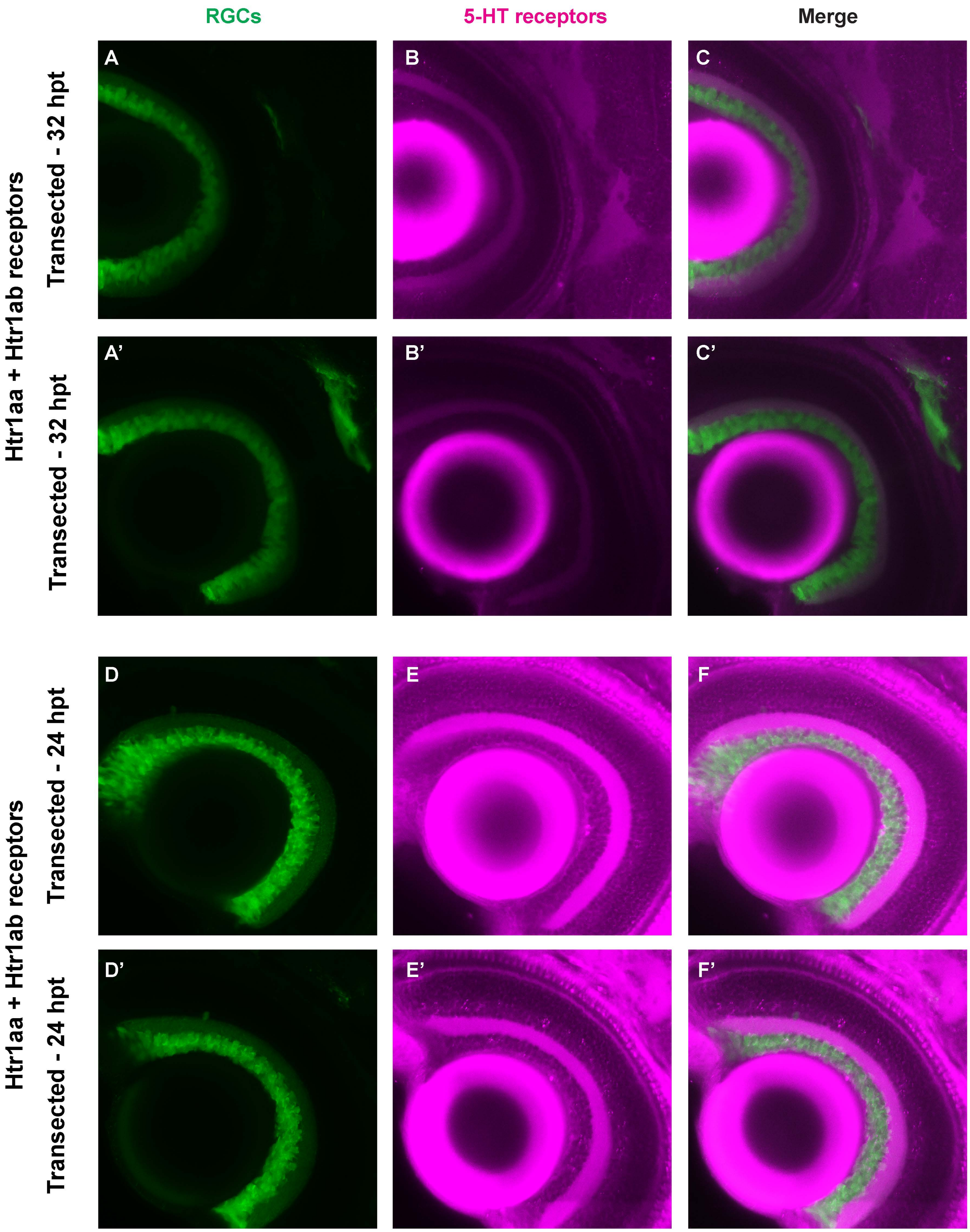

Saied-Santiago *et al.*, Supplemental Figure 2

Fig. S2. No staining observed in RGCs after incubating fixed larvae with *htr1aa* and *htr1ab* probes at 32 hpt.

(A - F) Representative images of retinas from *Tg(isl2b:GFP)* larvae with transected optic nerves at 32 hpt (A – C', n=2) or 24 hpt (D – F', n=2) stained with an *in situ* hybridization HCR probe mix for *htr1aa* and *htr1ab* (magenta). Merged maximum Z-projections are shown in (C-C' & F-F'). Images shown are maximum Z-projections, (32 um), 40X objective.

Development • Supplementary information

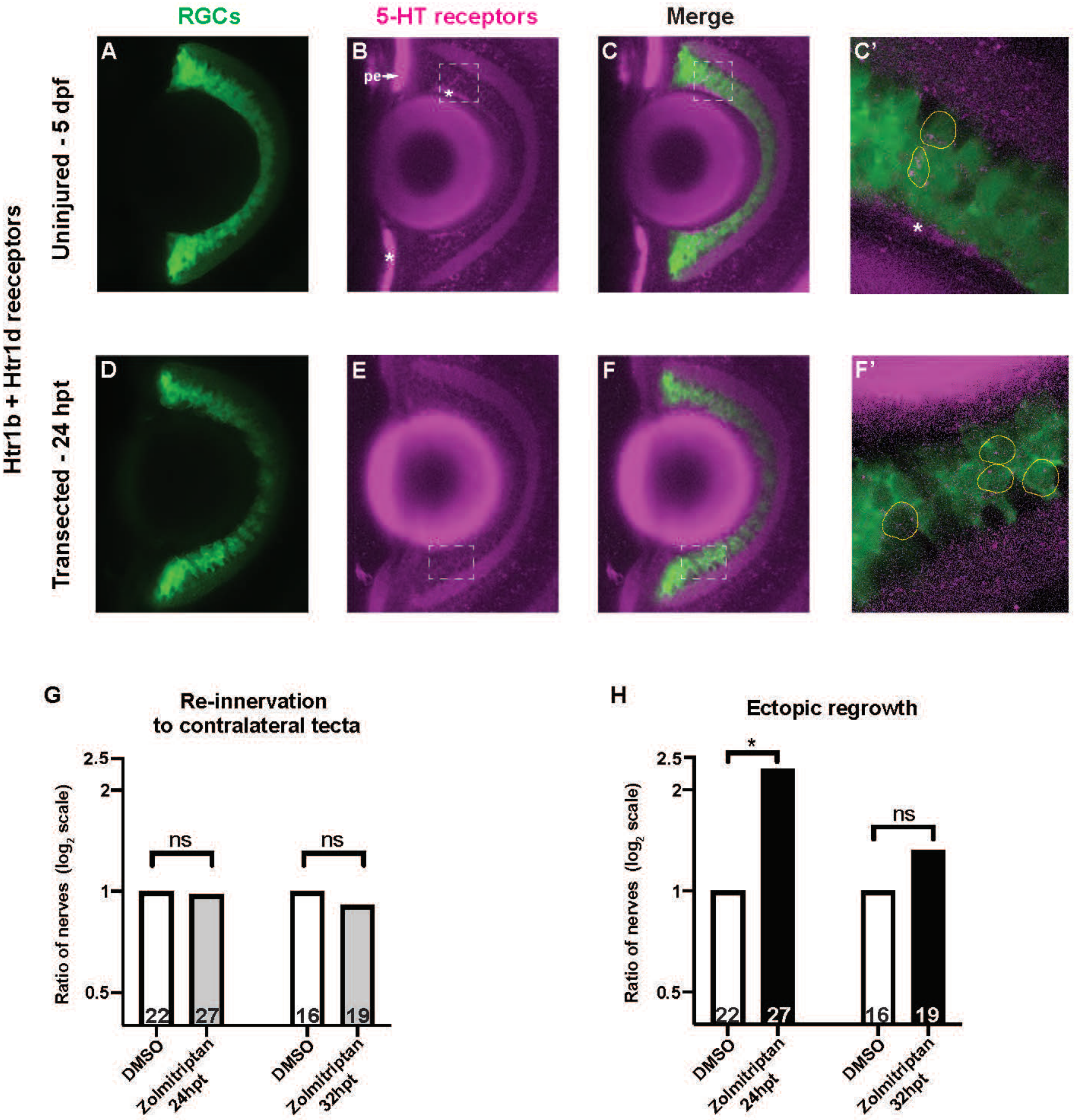

Fig. S3. 5-HT1B and 5-HT1D receptor genes are expressed in RGCs at pre-transection and during optic nerve regeneration.

(A - F) Representative images of retinas from Tg(isl2b:GFP) 5 dpf uninjured larvae (A-C', n=8) or larvae with transected optic nerves (D-F', n=3) at 24 hpt stained with an in situ hybridization HCR probe mix for htr1b and htr1d (magenta). Images shown are a maximum Z-projection of 10 optical sections (A-C) and 6 optical sections (D-F)(32 um), 40X. Dashed white boxes in (Figure B-C) is the area enlarged 1.5X in (C'), a merged maximum Z-projection of 15 optical sections (0.1 um). In (C'), yellow dashed lines outline cell bodies with mRNA expression. The brightness of the green channel was adjusted for better visualization of 5-HT1 receptor genes inside RGC neurons. White asterisks depict unspecific staining. The retinal pigmented epithelium (pe) was nonspecifically labeled by the amplifier with Alexa Fluor 546.

Development • Supplementary information

Dashed white boxes in (Figures E-F) is the area enlarged 1.5X in (F'), a merged maximum Z projection of 6 optical sections (0.1 um). In (F'), yellow dashed lines outline cell bodies with mRNA expression. The brightness of the green channel was adjusted for better visualization of 5-HT1 receptor genes inside RGC neurons.

(G - H) Quantification of optic nerve regeneration phenotypes at 48 hpt in Tg(isl2b:GFP) larvae treated with DMSO 0.3% or 50 uM of the agonist Zolmitriptan at 24 and 32 hpt. Bar graphs and ratios observed were calculated as detailed in Figure 5D and in Material and Methods. Optic nerve regrowth phenotypes analyzed include optic nerve re-innervation to contralateral tecta (G, gray bars in Zolmitriptan treated) and ectopic axonal regrowth (H, black bars in Zolmitriptan treated). Data showing 50 uM Zolmitriptan at 24 hpt are identical to Figures 5D-E and shown here for visual comparison only. Statistics determined using the two-tailed Fisher's exact test. Asterisks in figures represent statistical significance: * $P < 0.05$; ns, not significant. n=22 and n=27, for nerves treated with DMSO and Zolmitriptan at 24 hpt, respectively. n=16 and n=19, for nerves treated with DMSO and Zolmitriptan at 32 hpt, respectively.

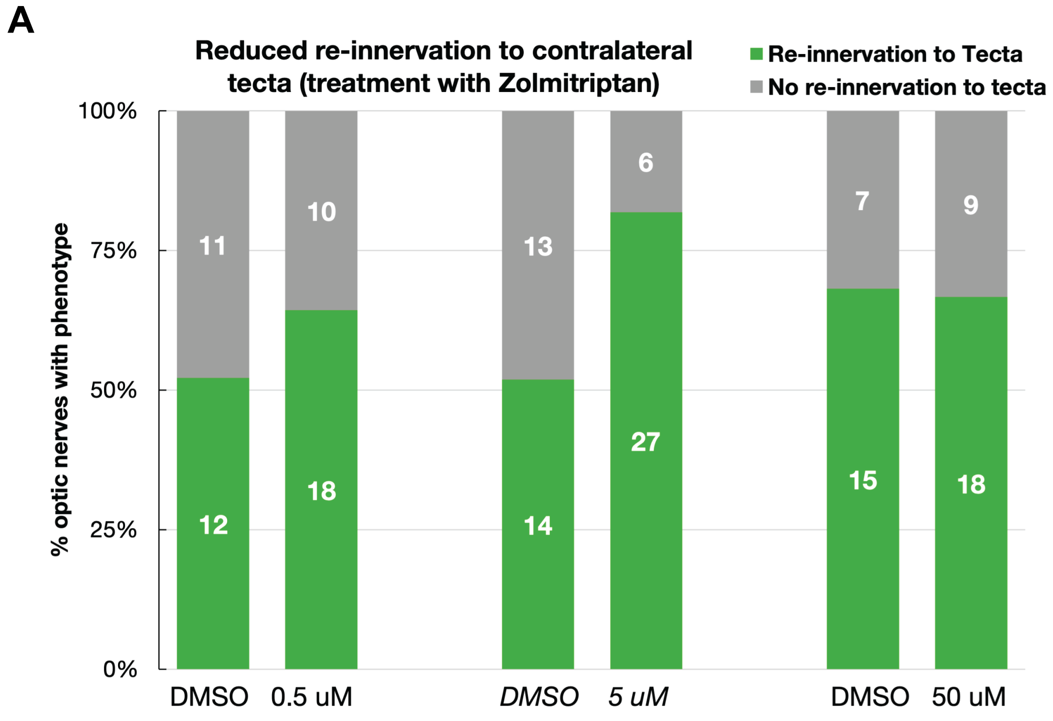

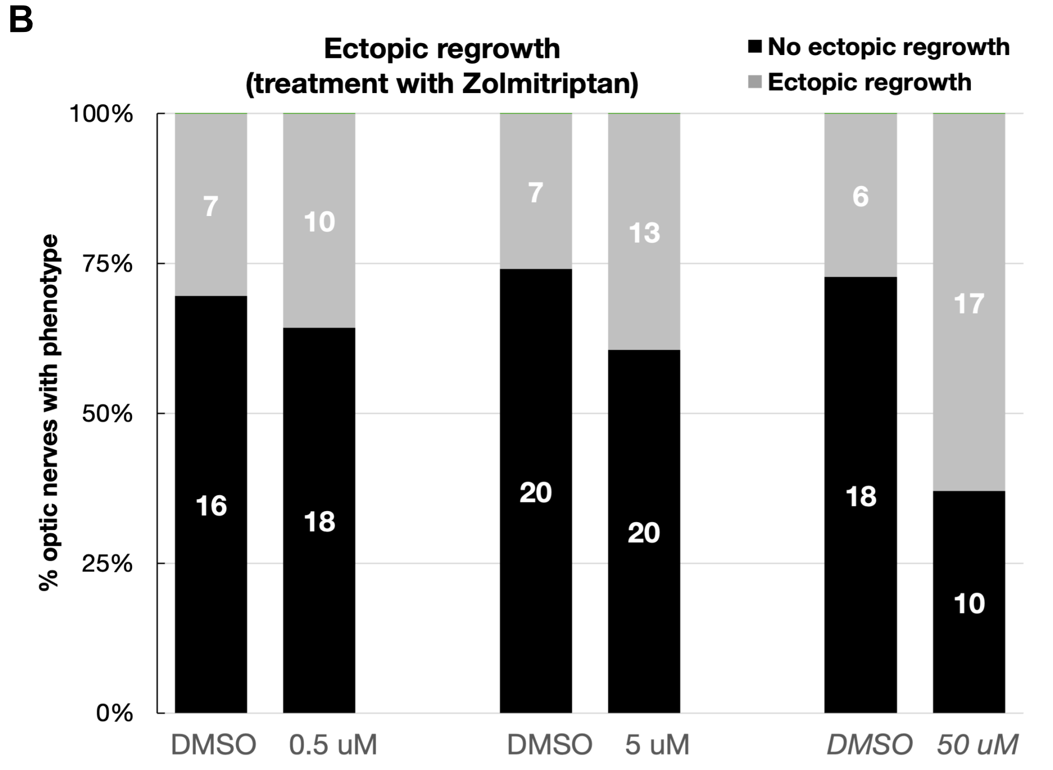

Development • Supplementary information

Fig. S4. Additional graphs and data of larvae treated with Zolmitriptan during optic nerve regeneration.

(A) Stacked bar graphs showing the percentage of optic nerves that re-innervated or did not re-innervated the contralateral tecta by 48 hpt in (TgIsl2b:GFP) larvae treated at 24 hpt with DMSO 0.3%, 0.5 of the agonist Zolmitriptan, 5 uM of Zolmitriptan and 50 uM of Zolmitriptan. Green bars show the percentage of optic nerves that re-innervated the contralateral tectum. Black bars show the percentage of optic nerves that did not re-innervated the contralateral tectum. Numbers inside the bars correspond to the number of optic nerves with a given phenotype. Data displayed was obtained from the same experimental groups analyzed in Figure 5D. n=23 and n=28 for nerves treated with DMSO and 0.5 uM Zolmitriptan, respectively; n=27 and n=33 for nerves treated with DMSO and 5 uM Zolmitriptan, respectively; and n=22 and n=27 for nerves treated with DMSO and 50 uM Zolmitriptan, respectively. Please refer to Figure 5D for details on statistical significance. Italicized text highlights groups showing statistical significance.

(B) Stacked bar graphs showing the percentage of optic nerves with ectopic regrowth by 48 hpt in (TgIsl2b:GFP) larvae treated at 24 hpt with DMSO 0.3%, 0.5 of the agonist Zolmitriptan, 5 uM of Zolmitriptan and 50 uM of Zolmitriptan. Black bars show the percentage of optic nerves with no ectopic regrowth. Gray bars show the percentage of optic nerves with ectopic regrowth. Numbers inside the bars correspond to the number of optic nerves with a given phenotype. Data displayed was obtained from the same experimental groups analyzed in Figure 5E. n=23 and n=28 for nerves treated with DMSO and 0.5 uM Zolmitriptan, respectively; n=27 and n=33 for nerves treated with DMSO and 5 uM Zolmitriptan, respectively; and n=22 and n=27 for nerves treated with DMSO and 50 uM Zolmitriptan, respectively. Please refer to Figure 5E for details on statistical significance. Italicized text highlights groups showing statistical significance.

Table S1. Molecular pathways tested for role in optic nerve regeneration.

Available for download at
https://journals.biologists.com/dev/article-lookup/doi/10.1242/dev.204334#supplementary-data

Table S2. Raw data of optic nerve regeneration experiments.

Available for download at
https://journals.biologists.com/dev/article-lookup/doi/10.1242/dev.204334#supplementary-data
